# Conventional protein kinase C in the brain: repurposing cancer drugs for neurodegenerative treatment?

**DOI:** 10.1042/NS20210036

**Published:** 2021-10-08

**Authors:** Gema Lordén, Alexandra C. Newton

**Affiliations:** Department of Pharmacology, University of California, San Diego, La Jolla, CA 92037, U.S.A.

**Keywords:** Alzheimer's Disease, Enzyme mutation, neurodegeneration, protein kinase C, Signal transduction, Spinocerebellar ataxia

## Abstract

Protein Kinase C (PKC) isozymes are tightly regulated kinases that transduce a myriad of signals from receptor-mediated hydrolysis of membrane phospholipids. They play an important role in brain physiology, and dysregulation of PKC activity is associated with neurodegeneration. Gain-of-function mutations in PKCα are associated with Alzheimer’s disease (AD) and mutations in PKCγ cause spinocerebellar ataxia (SCA) type 14 (SCA14). This article presents an overview of the role of the conventional PKCα and PKCγ in neurodegeneration and proposes repurposing PKC inhibitors, which failed in clinical trials for cancer, for the treatment of neurodegenerative diseases.

## Introduction

Protein phosphorylation is an important cellular regulatory mechanism that controls many aspects of cell fate. It is one of the most important post-translational modifications in the cell as many enzymes, receptors, and transcription factors are activated or deactivated by phosphorylation or dephosphorylation events. The balance between these two processes, catalyzed by kinases and phosphatases respectively, is critical for maintaining cellular homeostasis, and is often deregulated in diverse pathophysiological conditions. The family of nine protein kinase C (PKC) isozymes is crucial in tuning this equilibrium in the cell. These isozymes transduce signals from extracellular stimuli, with an important role in numerous cellular processes such as apoptosis, migration, and proliferation [1,2]. Since their precisely controlled activity finely balances cell survival versus cell death pathways, their deregulation is associated with a variety of diseases, including metabolic disorders, cancer, and neurodegeneration [1,3–5]. This review focuses on recent findings on aberrant PKC function in neurodegeneration.

## PKC maturation and signaling

PKC isozymes belong to a family of serine/threonine kinases categorized into three subclasses: conventional (α, β, and γ), novel (δ, ε, η, and θ), and atypical (ζ, λ/ι) [1,6]. All PKC isozymes comprise an N-terminal regulatory moiety that allosterically regulates a C-terminal catalytic moiety. The catalytic moiety is well conserved among the different PKC isozymes and consists of a kinase domain and a C-terminal tail. Within this region, there are three phosphorylation sites vital to PKC maturation: the activation loop, the turn motif, and the hydrophobic motif [7]. The regulatory moieties differ between the different PKC subclasses depending on the second messengers they bind for activation. Specifically, the regulatory moiety of conventional PKC isozymes contains two tandem diacylglycerol (DAG)-binding C1 domains, and a Ca^2+^-binding C2 domain, allowing them to respond to both DAG and Ca^2+^; novel PKC isozymes also have DAG-binding C1 domains, but their C2 domain is not a Ca^2+^ sensor, so they respond to only DAG; atypical PKC isozymes have one C1 domain that does not bind DAG and a PB1 protein interaction domain, and their activation depends on protein:protein interactions [8]. Of all the DAG-regulated PKC isozymes, PKCα is the only one that is established to have a functional type I PDZ-binding motif, a four amino acid segment (QSAV) located in its C-terminus, which mediates its interaction with PDZ-domain containing binding partners such as synapse-associated protein 97 (SAP97), post-synaptic density protein 95 (PSD95), and protein interacting with C kinase 1 (PICK1) [9,10].

Following their biosynthesis, conventional PKC isozymes are processed by a series of constitutive phosphorylations that are necessary for the enzymes to adopt an autoinhibited and stable conformation [7]. These priming phosphorylations are mediated by mTORC2 at a recently identified TOR Interaction Motif and adjacent turn motif on the C-tail, promoting phosphorylation by the phosphoinositide-dependent kinase 1 (PDK1) at the activation loop, in turn triggering an intramolecular autophosphorylation at another key regulatory site in the C-tail, the hydrophobic motif [11]. In the autoinhibited state, PKC is relatively resistant to dephosphorylation and subsequent degradation, and it has a half-time on the order of days [12]. This autoinhibited enzyme is transiently and reversibly activated by second messengers that recruit PKC to the plasma membrane where it is locked in an open and active conformation that can phosphorylate substrates and propagate downstream signaling. In contrast with the autoinhibited (closed) conformation, PKC in the active (open) conformation is sensitive to dephosphorylation and subsequent degradation [13]. Thus, to avoid degradation, active PKC quickly reverts to its inactive, closed conformation once the second messengers return to basal levels. However, treatment of cells with phorbol esters or bryostatins, strong PKC activators which are not readily metabolized, cause PKC to be trapped in an open conformation resulting in initial acute activation followed by dephosphorylation and chronic down-regulation of the enzyme [14]. This down-regulation of chronically activated PKC has not only been observed in cellular studies, but also in peripheral blood monocytes from advanced metastatic cancer patients undergoing prolonged bryostatin treatment [14,15].

The first step in down-regulation of PKC is the dephosphorylation of the hydrophobic motif by the PH domain leucine-rich repeat protein phosphatase, PHLPP, an enzyme that plays a key role in setting the steady-state levels of PKC [13,16]. Dephosphorylation of the hydrophobic motif promotes protein phosphatase 2A (PP2A)-dependent dephosphorylation of the turn motif and activation loop, promoting ubiquitination and proteasomal, or in some cases, lysosomal degradation [17–19]. Curiously, occupancy of the active site with ATP-competitive inhibitors, or substrates, renders PKC resistant to dephosphorylation and hence down-regulation [20–23]. Phosphorylation controls the steady-state levels of PKC, in turn setting the amplitude of second messenger-dependent signaling.

**Figure 1:**
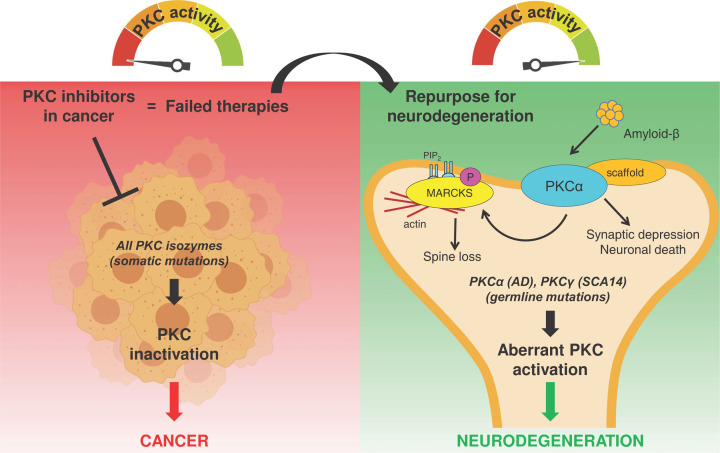
Importance of balancing PKC in the cell Left: Loss-of-function somatic mutations in all PKC isozymes have been associated with cancer. PKC inhibitors used in cancer clinical trials have failed, likely because PKC should be restored instead of being inhibited in this disease. Right: Germline mutations in PKCγ are associated with spinocerebellar ataxia and gain-of-function germline mutations in PKCα are associated with Alzheimer’s disease. Amyloid-β exposure leads to aberrant PKC function in neurons causing synaptic depression, neuronal death, and spine density loss. Repurposing of PKC inhibitors that failed in cancer clinical trials could be an effective therapeutic strategy to treat neurodegenerative disorders in which PKC is overactive. Figure created with Biorender.com

Conventional PKC isozymes are highly enriched in brain, with PKCγ found predominantly in the Purkinje cells in the cerebellum [24]. However, since the purification and characterization of PKC from brain tissue in the seventies by Nishizuka and co-workers [25,26], research on these enzymes has focused primarily on their role in oncogenesis. This is due in large part to the establishment, in the early eighties, of a paradigm for PKC as an oncoprotein based on its identification as the main receptor for the potent, tumor-promoting phorbol esters [27–29]. However, this dogma has been reversed in recent years after identifying that cancer-associated mutations of PKC are generally loss-of-function [30]. Furthermore, the steady-state protein levels of specific conventional PKC isozymes correlate with greater survival in cancers such as pancreatic cancer, colorectal, and non-small cell lung carcinoma [16,31–35]. Reframing PKC as a tumor suppressor would explain why the use of PKC inhibitors in cancer clinical trials have not only failed but in some cases, have even worsened patient outcome [36], supporting the hypothesis that decreased PKC activity promotes cellular growth and survival (Figure 1).

## Conventional PKC in the brain

As reviewed in more detail recently (the reader is referred to [5]), conventional PKC isozymes regulate many forms of synaptic plasticity in the brain, including long-term depression and long-term potentiation [5,37]. Members of this family of kinases phosphorylate diverse substrates that modulate neurotransmitter release, including ion channels, transporters, and G protein-coupled receptors [38–40]. Additionally, PKCα plays a critical role in regulation of the cytoskeleton by its the phosphorylation of myristoylated alanine-rich C-kinase substrate (MARCKS), one of the earliest identified PKC substrates [41–44] and growth-associated protein 43 (GAP43) [44,45], to trigger actin depolarization and dissociation of long actin filaments, respectively. PKC is also involved in the phosphorylation of tau, a microtubule-associated protein that controls microtubule dynamics [46,47]. PKC has also been reported to modulate the α-secretory proteolytic processing of amyloid precursor protein (APP) [48,49], a transmembrane protein that is mainly expressed in neuronal and glial cells [50]. The finding that PKC modulates synaptic plasticity, cytoskeleton dynamics, and APP processing poises this kinase as a central player in brain homeostasis, and dysregulation of its activity will result in diverse brain pathologies.

## Conventional PKCs in neurodegeneration

Conventional PKC isozymes are emerging as actionable targets in neurodegenerative diseases. Their enhanced activity is associated with increased risk of cerebral infarction, Alzheimer’s disease (AD), and spinocerebellar ataxia (SCA) [1]. How increased PKC activity contributes to neurodegeneration has recently come into sharp focus and will be discussed in detail in the following sections.

## PKC in AD

AD is the most prevalent neurodegenerative disorder, affecting 47 million people worldwide, and it is characterized by synaptic depression, aberrant neuronal activity, and impaired cognition [51–53]. Two of the major neuropathologic features of the disease are the presence in the brain of amyloid-β (Aβ) plaques generated by the sequential miscleavage of the APP by β- and γ-secretases [54,55] and an organized pattern of neurofibrillary tangles mainly composed of hyperphosphorylated tau aggregates [56]. Despite extensive studies implicating Aβ and tau in the detrimental effects observed during neurodegeneration associated with AD, the mechanisms by which Aβ leads to synaptic depression are yet to be fully elucidated. Nonetheless, targeting Aβ plaques forms the basis for the 2021 approved therapy (Aducanumab); this is the first approved therapy for AD since 2003, and the first treatment directed at the presence of Aβ plaques [57]. Despite this newly approved treatment, understanding the precise signaling mechanisms deregulated in AD will still be necessary to develop more therapies to help combat this devastating disease.

PKC has been implicated in both tau hyperphosphorylation and Aβ pathology [58], but conflicting roles have been attributed to PKC during Aβ-mediated pathogenesis. Early studies proposed pharmacological activation of PKC for the treatment of AD [59,60]. Treatment of APP-expressing cells with phorbol esters was shown to increase the non-pathogenic processing of APP by enhancing APP cleavage by α-secretases, ultimately reducing the production of pathogenic Aβ peptides [59–62]. Furthermore, antisense-mediated loss of PKCα in SH-SY5Y neuroblastoma cells was reported to impair this induction of non-pathogenic APP [63,64]. However, as discussed above, prolonged activation of PKC by phorbol esters causes a paradoxical loss of function by promoting its degradation [14], so it remains to be established whether the activation or down-regulation of PKC contributed to this effect. Moreover, different PKC isozymes have different roles in the brain [65–69], so targeting specific isozymes would be key to developing successful therapies.

More recent studies converge on enhanced PKC signaling as a key contributor to the development of AD. One of the most studied PKC isozymes in this context is the conventional PKCα, and mounting evidence points towards its unique PDZ-binding motif mediating its role in neurodegeneration [66,70]. This PDZ-binding motif directs PKCα to scaffolds such as PICK1 [10]. Genetic deletion of PICK1 or pharmacological inhibition of its interaction with glutamate ionotropic receptor α-amino-3-hydroxy-5-methyl-5-isoxazolepropionic acid (AMPA) type subunit 2 (GluR2) reverses the synaptic depression triggered by Aβ [70–72]. AMPA receptors (AMPARs) are tetrameric glutamate receptors that are responsible for the immediate postsynaptic response to glutamate [73]. These receptors mediate excitatory synaptic transmission, and the synaptic depression triggered by Aβ could be caused by the activation of kinases that target AMPARs such as PKCα, which phosphorylates S880 on GluR2 [74] altering the recycling of GluR2 [75]. Electrophysiological studies have established that PKCα is necessary for Aβ-mediated synaptic depression by a mechanism that depends on the PDZ ligand of PKCα [66]. Scaffolding proteins such as PICK1, SAP97, and PSD95 bind the PDZ ligand of PKCα, suggesting that PDZ-mediated interaction with these scaffolds is necessary for the detrimental effects caused by PKCα [9,10]. Taken together, these studies establish an Aβ-PKCα-PICK1 signaling axis that drives synaptic depression and underscores the importance of PKCα in AD.

The identification in 2015 of gain-of-function rare variants of PKCα that co-segregate with late-onset AD (LOAD), in families with no other genetic risk factors, provided clear support that enhanced PKCα signaling is causative in AD [66]. Cellular studies of three such mutants revealed that these mutations caused a modest increase in the agonist-evoked activation of PKCα without altering activation kinetics or autoinhibition [66]. Biochemical analysis of a variant identified in multiple families (M489V, near the active site) revealed that it enhances the catalytic rate of the enzyme without altering the on/off dynamics or autoinhibitory constraints [76]. This provides a unique mechanism to amplify PKCα signaling without rendering the enzyme sensitive to down-regulation; this contrasts with ‘activating’ cancer-associated mutations in PKC that impair autoinhibition, ultimately causing their down-regulation and paradoxical loss-of-function [12]. Thus, the M489V PKCα variant leverages a small increase in catalytic efficiency to drive enhanced activity without altering the stability of PKCα [76]. Analysis of whole brain from a mouse model harboring the PKCα-M489V AD-associated variant revealed that it is indeed more active than wildtype PKCα, as reflected by the increased phosphorylation of MARCKS, a major downstream target of this kinase [76]. The mutant PKCα-M489V was also expressed at the same steady-state levels as wildtype PKCα in the brain [76]. While it is possible that the mechanisms by which distinct mutations on PKCα contribute to AD differs from one to the next, the simplest explanation is that they all increase the agonist-evoked signaling by unique mechanisms that do not alter the steady-state levels of PKCα. This small increase in agonist-dependent signaling in a low-turnover cell type, such as neurons, likely results in damage that contributes to cumulative pathology as patients age.

In addition to targeted studies, unbiased phosphoproteomic studies also identify elevated PKC signaling associated with neurodegeneration [77–79]. Using phospho-mass spectrometry, Tagawa et al*.* identified the common core signaling network that is deregulated in human AD patients and several AD mouse models [77]. The largest protein network hub identified in this study was regulated by PKC, indicating that PKC has a large role in early deregulation of the phosphoproteome in multiple models of AD [77]. MARCKS and Marcks1, known targets of PKC, were among the AD core phosphoproteins identified [77]. More recently, two different phosphoproteomic studies of temporal and frontal cortex of AD patient brains also identified hyperphosphorylation of MARCKS and overactivity of PKC in the AD kinase network [78,79]. Curiously, phosphorylation of PKCα at T228 was enhanced in AD brains compared with control brains [79]. This site remains functionally uncharacterized, and it would be of high interest to determine its impact on PKCα stability or activity. Furthermore, one of the phosphoproteomics analyses of AD brains identified an increase in phosphorylation of T150 on MARCKS, a site that is not known to be phosphorylated by PKC and is, instead, predicted to be phosphorylated by extracellular signal-regulated kinase (ERK) [78,80]. However, phosphorylation at this site likely occurs downstream of PKC [81–83]. Overall, these unbiased studies identify enhanced PKC signaling output, and particularly hyperphosphorylation of the substrate MARCKS, in the development of this detrimental neurodegenerative disease.

In addition to MARCKS, GAP43 and spectrin α chain (SPTA2) have also been identified as critical targets of overactive PKC that contribute to the early and middle stages of AD development [77,78]. These PKC substrates are abundant in the brain and are located in the plasma membrane where they play a major role in the maintenance of actin filament cross-linking [41,45,84]. Their hyperphosphorylation disrupts cytoskeletal function, which is critical in the initiation of synapse pathology that results in AD pathogenesis. Identification of MARCKS as the most important candidate for the early stages of synapse-related AD pathology is supported by earlier studies showing that its phosphorylation at specific PKC sites promotes spine density loss and shrinkage, that is concomitant with a reduction in F-actin content [85]. This suggests that the deleterious effects of PKC on synaptic plasticity may, in part, result from excessive phosphorylation of MARCKS, leading to a disruption of the normal stability and shape of the dendritic spines in mature neurons.

Results described here suggest that the complimentary use of different unbiased approaches such as large-scale genome-wide association studies (GWASs) and phosphoproteomics are key to uncovering the deregulated mechanisms underlying AD. Furthermore, the identification of rare variants that increase the risk of developing AD pathophysiology provides invaluable clues as to the mechanisms underlying the pathogenesis and have been essential to making progress in the field; however, we note that such approaches have not identified a single specific gene responsible for causing LOAD [86–88]. The importance of this technology is illustrated by the discovery of AD-associated mutations on *APOE* and *TREM2* genes, which unveiled new mechanisms and pathways altered in the disease that can lead to the development of new agents to treat the disease [89–91]. Furthermore, the importance of PKC in the brain is not only illustrated by the discovery of AD-linked PKCα variants that enhance kinase output in the cell, but also reflected by the identification of AD-associated mutations in another PKC isozyme that belongs to the novel subfamily, PKCη [92]. Uncovering whether PKCη variants display similar anomalous effects on kinase signaling and subsequent neurogenerative features would be of interest to further characterize PKC function during the development of AD.

## PKCγ in SCA

Another neurodegenerative disease associated with an overly active PKC isozyme is SCA, a hereditary disorder characterized by Purkinje cell degeneration in the cerebellum [93,94]. SCAs are classified into different subtypes according to the mutated gene responsible for the disease [94]. Specifically, point mutations identified in the *PRKCG* gene, which encodes for the conventional protein kinase C γ (PKCγ), are responsible for causing spinocerebellar ataxia type 14 (SCA14) [95–97].

Over 50 mutations in PKCγ have been identified as causative in SCA14, and numerous mechanisms including increased PKCγ activity [65,98,99], protein aggregation [100–102], enzyme mislocalization [65], and altered proteasomal degradation [101], have been proposed to account for the pathology. Mutations known to cause SCA14 are located through all the domains of PKCγ but curiously, most mutations occur in the DAG-sensing C1B domain [65,99]. Despite multiple studies performed to understand the underlying mechanisms that induce SCA14, a common mechanism has not yet been found. However, animal model studies provide important insight. Notably, mice engineered to have an SCA14-associated mutation in the pseudosubstrate that results in constitutive activity (A24E) display an ataxic phenotype [103]. Although this mutation destabilizes the ‘open’ PKC resulting in an approximately ten-fold reduction in steady-state levels of the mutant PKC, the deregulated activity is sufficient to cause an ataxic phenotype. PKCγ null mice do not develop ataxia [104–106], suggesting that it is not loss of PKCγ function driving the pathology. Treatment of isolated Purkinje cells with PKC inhibitors prevents cell death and results in Purkinje neurite extension, whereas treatment with PKC activators results in Purkinje cell death [107,108]. These data suggest that the development of agents that inhibit PKCγ activity would be instrumental to prevent the cerebellar dysfunction observed in patients with SCA14.

Gain-of-function mutations in PKCα and in PKCγ are associated with neurodegenerative disease. For both diseases, enhanced signaling over the lifetime of a long-lived neuron could accumulate damage. The development of specific inhibitors or use of antisense oligonucleotides specific for each isozyme would result in highly specific and innovative therapeutic strategies for the treatment of neurodegenerative diseases.

## Concluding remarks

PKC is emerging as a biomarker and therapeutic target in neurodegenerative disease. In particular, the Ca^2+^-regulated PKC isozymes that are found in the brain, PKCα and PKCγ, are associated with AD and SCA, respectively, promoting synaptic loss and neuronal death in these different neurological disorders. Accumulating evidence now suggests that inhibiting PKC could be a viable strategy to reverse or slow down the neurodegeneration associated with AD. Whereas inhibition of PKC using pharmacological inhibitors or aprinocarsen, a PKCα antisense oligonucleotide, failed in clinical trials for cancer, these same molecules may be more effective if repurposed for AD [36]. Indeed, the use of specific PKC antisense oligonucleotides to reduce PKC is an attractive potential treatment of neurodegenerative diseases since antisense strategies are already successfully used to ameliorate clinical manifestation of spinal muscular atrophy [109], to decrease superoxide dismutase 1 in order to treat amyotrophic lateral sclerosis [110], and to reduce leucine-rich repeat kinase 2 (LRRK2) protein levels in Parkinson’s disease treatment [111], among others [112,113]. Antisense oligonucleotides can be restricted to the central nervous system to reduce protein levels exclusively in the brain, providing a precision medicine approach which would be valuable for neurodegenerative diseases in which PKC activity should be reduced to restore homeostasis. Specificity in inhibition can be leveraged by targeting the unique PDZ ligand interactions of PKCα at the synapse. Indeed, small molecule binders of the PDZ domain of PICK1, which scaffolds PKCα, has previously been shown to prevent Aβ-induced synaptic depression [10,72]. Importantly the amplitude of PKC signaling in AD and ataxia only needs to be tuned to homeostatic levels, not abolished. This slight tuning of activity would avoid detrimental effects associated with loss of activity observed in cancer. Our detailed understanding of the molecular mechanisms controlling PKC poise it as an attractive and druggable target. The discovery of PKC in the brain 40 years ago was followed by extensive studies outside the brain, yet its aberrant function in the brain may be its clearest disease role.

## Abbreviations

AD: Alzheimer’s disease
AMPA: α-amino-3-hydroxy-5-methyl-5-isoxazolepropionic acid
AMPAR: AMPA receptor
APP: amyloid precursor protein
Aβ: amyloid-β
DAG: diacylglycerol
GAP43: growth-associated protein 43
GluR2: glutamate ionotropic receptor AMPA type subunit 2
LOAD: late-onset Alzheimer’s disease
MARCKS: myristoylated alanine-rich C-kinase substrate
PICK1: protein interacting with C kinase 1
PKC: protein kinase C
PP2A: protein phosphatase 2A
PSD95: post-synaptic density protein 95
SAP97: synapse-associated protein 97
SCA: spinocerebellar ataxia
